# Sold Out!

**Published:** 1854-02

**Authors:** 


*Sold Out!*—The private reputation, as well as that derived from a former connection with various medical schools, of Ex-Prof. James McClintock, has been put up at public sale. A druggist firm in New York are the happy purchasers of this extremely valuable property.

The ostensible value received in this transaction, consists of a number of recipes for pills, cough syrups, and the like, which are now to be added to the armamentaria of "popular" medicine. These formulæ are, as we are informed, the result of vast experience, and profound science, and Dr. McClintock thus disposes of them, to rescue himself from the necessity of answering the myriad of letters from his former students, and the profession at large, inquiring their composition.

The real *quid pro quo* in the bargain, is the liberty to use the name and reputation of one who *might* have been a man, for the base purposes of open and shameful quackery. This is the only instance wherein a somewhat noted teacher of medicine has thus degraded himself. Had Dr. McClintock only sold the recipes, it would have been simply disgraceful; but when he sells himself—the little that was left of his former reputation—and, by implication, the good names of those formerly associated with him, there is no adjective which can describe the transaction.

The doctrine of original sin, and innate depravity are, in this, fully established. There are no other means of accounting for such a wofully mean exodus from an honorable profession. Men usually fall slowly; they come down, step by step, in their downward progress. But here, so far as we can see, the descent is sudden. The doctor falls to the base earth of quackery like a bear from a tree, and, apparently, with as little discomposure to his own *bon hommie.*

It cannot be that there is any great cause for mourning in this event. We do not look upon it as any indication of decreasing respectability in the profession. The Rev. Dr. Dodd committed forgery, but that has never been laid to his cloth, or referred to as a sign of the times. When a man by dishonesty, by intemperance, or other vicious habits, disgraces his associations, it is for the reason that we have assigned. The sin was in him. Circumstances, the ambitions of youth, the possession of talents, the influence of friends, may all make a man respectable for a season, but if he is naturally a quack, he herds at last with his fellows.

But, for the credit of the name of Professor, we hope that this speculation in the character and the names of those who have been so unfortunate as to be associated with its author, may fail, and that thus the public prints may not be daily trumpeting, at once, the fancied virtues of the medicines, and the disgrace of their compounder.

We have said more about this matter than its importance merits; but it was something new to see a man's character put up in pint and half-pint bottles, to be retailed in every country drug-store, and hawked about the land by itinerant peddlers. H.

